# Argon: Neuroprotection in *in vitro *models of cerebral ischemia and traumatic brain injury

**DOI:** 10.1186/cc8214

**Published:** 2009-12-17

**Authors:** Philip D Loetscher, Jan Rossaint, Rolf Rossaint, Joachim Weis, Michael Fries, Astrid Fahlenkamp, Yu-Mi Ryang, Oliver Grottke, Mark Coburn

**Affiliations:** 1 Department of Anesthesiology, University Hospital of the RWTH Aachen, Pauwelsstraße 30, 52074 Aachen, Germany; 2 Institute of Neuropathology, University Hospital of the RWTH Aachen, Pauwelsstraße 30, 52074 Aachen, Germany; 3 Department of Surgical Intensive Care, University Hospital of the RWTH Aachen, Pauwelsstraße 30, 52074 Aachen, Germany; 4 Department of Neurosurgery, University Hospital of the RWTH Aachen, Pauwelsstraße 30, 52074 Aachen, Germany

## Abstract

**Introduction:**

Recently, it has been shown in several experimental settings that the noble gases xenon and helium have neuroprotective properties. In this study we tested the hypothesis that the noble gas argon has a neuroprotective potential as well. Since traumatic brain injury and stroke are widespread and generate an enormous economic and social burden, we investigated the possible neuroprotective effect in *in vitro *models of traumatic brain injury and cerebral ischemia.

**Methods:**

Organotypic hippocampal slice cultures from mice pups were subjected to either oxygen-glucose deprivation or to a focal mechanical trauma and subsequently treated with three different concentrations (25, 50 and 74%) of argon immediately after trauma or with a two-or-three-hour delay. After 72 hours of incubation tissue injury assessment was performed using propidium iodide, a staining agent that becomes fluorescent when it diffuses into damaged cells via disintegrated cell membranes.

**Results:**

We could show argon's neuroprotective effects at different concentrations when applied directly after oxygen-glucose deprivation or trauma. Even three hours after application, argon was still neuroprotective.

**Conclusions:**

Argon showed a neuroprotective effect in both *in vitro *models of oxygen-glucose deprivation and traumatic brain injury. Our promising results justify further *in vivo *animal research.

## Introduction

The first biological effects of argon were demonstrated as early as 1939 [1]. Behnke et al. described the narcotic effects of argon as experienced by deep sea divers at high pressures. Half a century later Soldatov and co-workers [2] were the first to show argon's protective effects under hypoxic conditions. Thereafter, it was reported that argon shields hair cells from ototoxic process [3] and protects cell cultures from ischemia [4]. In contrast to argon, xenon's organ protective effects have been investigated in various settings and models, ranging from cell cultures to clinical trials. Xenon has proven to be a safe anaesthetic agent and xenon's organoprotective properties have been demonstrated in many fields [5-14].

Stroke and traumatic brain injury (TBI) are two very common causes of death and disability worldwide and create a significant economic and social burden [15-17]. While the acute treatment of stroke today is highly standardized and secondary prevention is effective, an efficient protection of the cells at risk in the penumbra is lacking. This is particularly evident in regard to TBI. Although an estimated 1.5 million people in the United States suffer from TBI annually [15,17] due to the diverse mechanisms of the initial trauma itself and the following molecular pathways, a specific treatment is still absent.

When compared to xenon argon has some conspicuous advantages: low cost; no narcotic effects at normobaric pressures. Yet, data on argon's effects on cells are sparse. Therefore, we tested the effect of argon in *in vitro *models that involved either a focal mechanical trauma or oxygen-glucose deprivation of cultured hippocampal slices.

## Materials and methods

All experiments were performed in compliance with the local Institutional Ethical Review Committee and have been approved by the animal protection representative at the Institute of Animal Research at the RWTH Aachen University Hospital, according to the German animal protection law §4, Section 3. Unless otherwise stated, all chemicals were obtained from PAA Laboratories GmbH (Pasching, Austria).

### Organotypic hippocampal slice cultures

Cultures were prepared as previously reported [18], with some modifications [8,19]. Briefly, brains from six-to-eight-day-old mice pups (C57BL/6N, Charles River Laboratories, Sulzfeld, Germany) were extracted and directly transferred to ice-cold preparation medium (Gey's balanced salt solution (Sigma-Aldrich, Munich, Germany), 5 mg/ml D-(+)Glucose (Roth, Karlsruhe, Germany), 0.1 Vol. % antibiotic/antimycotic solution (penicillin G 10,000 units/ml, streptomycin sulphate 10 mg/ml, amphotericin B 25 μg/ml). The hippocampi were rapidly removed from the brains, cut into 400 μm thick transverse slices with a McIllwain tissue chopper (The Mickle Laboratory Engineering Co. Ltd., Gomshall, UK) and arranged onto the membrane of a MilliCell tissue culture insert (MilliCell-CM, Millipore Corporation, Billerica, MA, USA). The inserts were placed in tissue culture plates (Sarstedt, Newton, MA, USA) and 0.8 ml growth medium (50% Eagle minimal essential medium with Earle's salts, 25% Hank's balanced salt solution (Sigma-Aldrich, Munich, Germany), 25% heat inactivated horse serum, 2 mM L-glutamine, 5 mg/ml D-glucose, 1% antibiotic/antimycotic solution and 50 mM HEPES buffer solution (Fluka, Buchs, Switzerland), titrated to pH 7.2) was inserted underneath the membrane. The hippocampal slice cultures were incubated for 14 days and growth medium was exchanged every third day.

### Oxygen glucose deprivation

After two weeks in culture the growth medium was exchanged with experimental medium (EM). EM was similar to growth medium but the horse serum was replaced in equal measure by Eagle minimal essential medium. Additionally, to allow fluorescence imaging, 4.5 μM propidium iodide (PI) (Sigma-Aldrich, Munich, Germany) was added and the slices were then incubated for 30 minutes at 37°C and 5% CO_2_. After baseline fluorescence imaging, oxygen-glucose deprivation (OGD) was accomplished as previously described [20,21] with minor modifications. First, 50 ml of glucose free experimental medium was saturated with 95% N_2_, 5% CO_2_. After replacing the culture medium with the oxygen-glucose deprived medium the plates were transferred into an airtight pressure chamber (volume = 750 ml) equipped with inlet and outlet valves. The chamber was immediately flushed with a humidified gas mixture (5% CO_2_, 95% N_2_) for two minutes at a flowrate of 2.5 l/min to ensure a >99% gas exchange in the chamber which was then sealed. After 30 minutes of oxygen-glucose deprivation at 37°C, the medium was replaced by EM containing glucose and 4.5 μM PI. Immediately after the slices were relocated to the pressure chamber, the chamber was flushed with the experimental gas mixture and sealed.

Slices incubated for 72 hours in 5% CO_2_, 21% O_2 _and 74% N_2 _were considered to be the OGD trauma control group. The negative control (no OGD) was subjected to the identical treatment. Yet the EM contained 5 mg/ml glucose and was saturated with 5% CO_2_, 21% O_2 _and 74% N_2 _and the pressure chamber was flushed with 5% CO_2_, 21% O_2 _and 74% N_2_. For experimental groups, the pressure chamber was flushed using a premixed argon gas mixture (5% CO_2_, 21% O_2_, x% Argon, [74-x]% N_2_; Air Liquide Santé International, Paris, France).

### Traumatic brain injury

All slices subject to traumatic brain injury were first incubated for 30 minutes at 37°C, 95% air and 5% CO_2 _in EM containing 4.5 μM PI. Following baseline fluorescence imaging the traumatic brain injury was generated using an apparatus designed as previously described [8,19,22]. It allowed dropping a stylus in a reproducible manner under stereomicroscopic supervision with a three-axis micromanipulator from a height of 7 mm with a force of 5.26 μJ onto the CA1 region of the hippocampal slices. The shape of the stylus was round to prevent piercing of the tissue. After traumatizing the CA1 region the medium was changed to EM, containing 4.5 μM PI. The cultures were then placed into the pressure chamber for 72 hours before the final imaging. The TBI-trauma control group (that is, traumatized) was incubated in an atmosphere of 5% CO_2_, 21% O_2 _and 74% N_2_. The negative control group, not subject to TBI-trauma, followed the identical cycle to the trauma group, but the pin was not dropped onto the slice. The pressure chamber for experimental groups contained 5% CO_2_, 21% O_2_, x% Argon and [74-x]% N_2_.

### Staining and microscopy

Propidium iodide is a fluorescent intercalating agent which is widely used to stain DNA [19,21,23]. While it is unable to penetrate viable cells it can diffuse into damaged cells when the membrane is disintegrated. Upon binding to the DNA it becomes highly fluorescent. PI fluorescence was observed with a fluorescence microscope (Zeiss Axioplan, Carl Zeiss MicroImaging GmbH, Jena, Germany) and a low-power 4× objective (Zeiss Achroplan 4×/0.10, Carl Zeiss MicroImaging GmbH, Jena, Germany) and recorded with a digital camera and appropriate software (SPOT Pursuit 4 MP Slider, Diagnostic Instruments Inc, Sterling Heights, MI, USA; MetaVue, Molecular Devices, Sunnyvale, CA, USA). The exposure time was adjusted to the fluorescence magnitude captured from a standard slide (Fluor-Ref, Omega Optical, Brattleboro, VT, USA) to accommodate for the mercury lamp's fluctuating intensity over time.

### Assessment of cell injury

**Figure 1:**
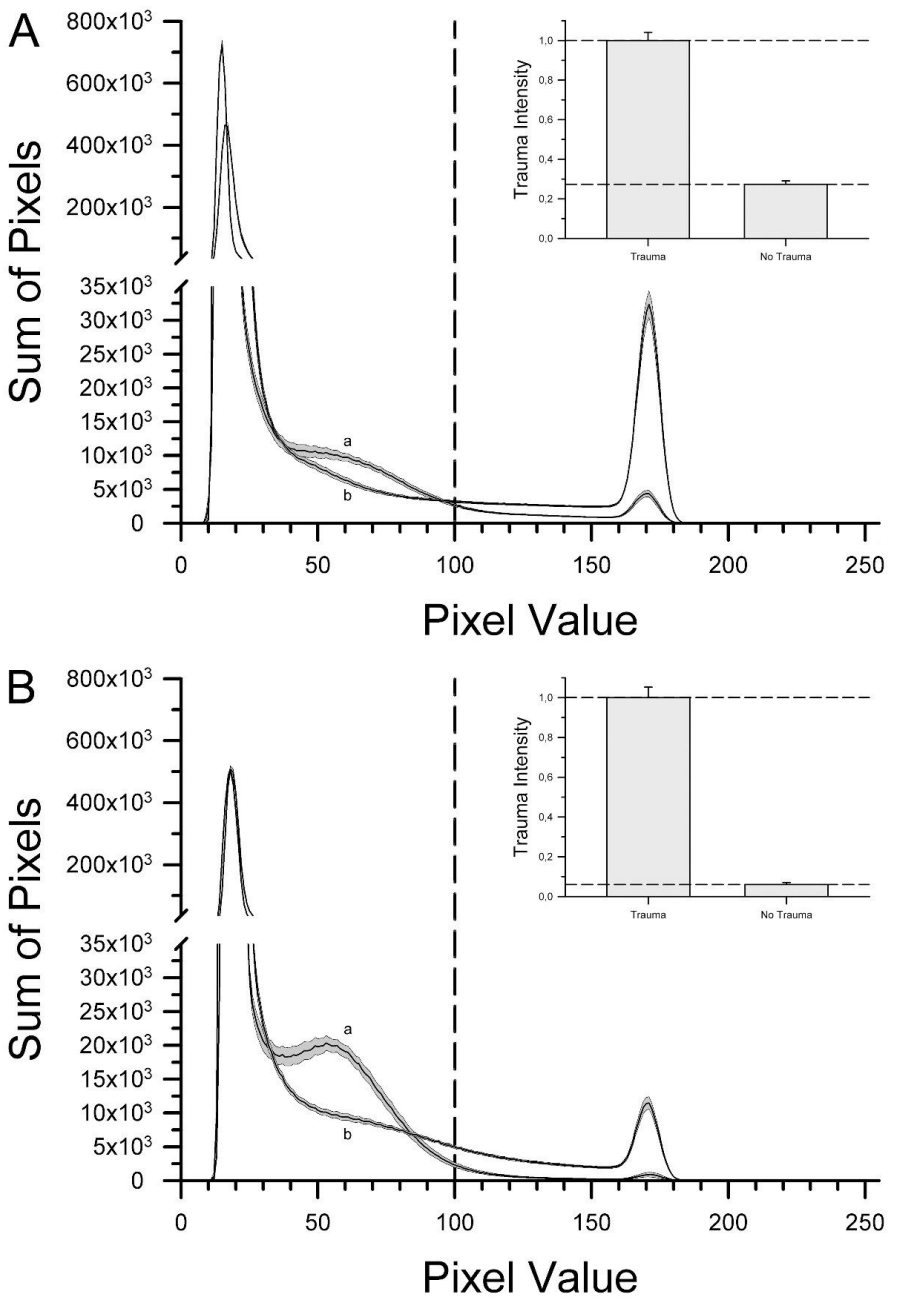
**Control data**. After preparation, 14 days of cultivation and baseline fluorescence imaging, slices were either impaired with oxygen-glucose deprivation (OGD) or traumatic brain injury (TBI) (see **panel A **or **B **respectively). For OGD, the slices were incubated in glucose free medium and transferred into an airtight anoxic chamber where they were incubated in an atmosphere of 95% N_2 _and 5% CO_2 _for 30 minutes. TBI was induced by the impact of a stylus onto the CA1 region of the hippocampus. After trauma, the slices were transferred to an airtight chamber and incubated in an atmosphere of 21% O_2_, 5% CO_2 _and 74% N_2_. The negative control groups' slices were subjected to the same treatment, except for the trauma. After 72 hours the damage was assessed by fluorescence imaging and pixel-based image analysis. In both panels, both curves labelled as *a *show the histogram of non-traumatized slices (OGD: n = 58 prepared from six mice; TBI: n = 35 prepared from six mice) after 72 hours. The middle line is the mean value; the upper and lower lines represent the upper and lower bounds of the SEM. Curves *b *present the histogram of traumatized slices (OGD: n = 71 prepared from eight mice; TBI: n = 39 prepared from six mice). The vertical dashed line is the applied threshold at a gray scale value of 100. The sum over all pixel values greater than this threshold were calculated for each group and defined as the trauma intensity. Inserts in panel A and B respectively present the controls normalized to the trauma groups.

The fluorescence images were digitalized at eight bit, allowing us to differentiate between a spectrum of 256 (from 0 to 255) grey scale levels. Damaged regions with a high PI uptake emitted at a high grey scale level, while vital regions showed only minor emissions. The red channel of each image was analyzed with ImageJ software (National Institutes of Health, Bethesda, MD, USA) [24]. ImageJ generated a histogram for each image which showed the absolute number of pixels with the same grey scale value. Histograms from non-traumatized slices (see Figure 1) showed that the vast majority of all pixels had grey scale values between 10 and 100, representing mostly background fluorescence. In contrast, traumatized slices showed, in addition to their background fluorescence, a well-defined peak between 160 and 185. As in previous publications [8,19,25,26] we established a threshold (in this instance at a grey scale value of 100) which proved to be valid to distinguish between traumatized and non-traumatized cells. The integration of all pixels exceeding the threshold therefore was a sound quantification of cell injury.

### Statistical Analysis

After measuring the induced cell damage for each slice, these results were combined for every experimental group and then normalized to the intensities of trauma in each trauma group (OGD or TBI, respectively). Mean values and standard errors of the means (SEM) were calculated with SPSS 16.0 (SPSS Inc., Chicago, IL, USA). Statistical significance was evaluated with SPSS using the one-way analysis of variance (ANOVA) with bonferroni post-hoc analysis; *P *≤ 0.05 was considered as statistically significant.

## Results

Tissue damage after 14 days of incubation was negligible (as has been shown before, see Rossaint et al. [19]) otherwise slices were excluded. A distinct pattern of distribution of pixel values was evident for traumatized and non-traumatized groups 72 hours after inducing the experimental injury. Both groups shared a certain background fluorescence level after OGD or TBI (Figure 1). Above a threshold of a grey scale value of 100, the traumatized groups showed a characteristic peak in fluorescence between pixel values of 160 to 185. Non-traumatized slices in both OGD- and TBI-models displayed a minor, but still detectable, rise in emission at a similar luminance. All pixels above the threshold were summarized for each group. The integral for OGD and TBI trauma control groups was set as one and the sums for all further groups were normalized to the OGD and TBI trauma control group respectively as a quantitative measure for trauma intensity (see inserts in Figure 1).

**Figure 2:**
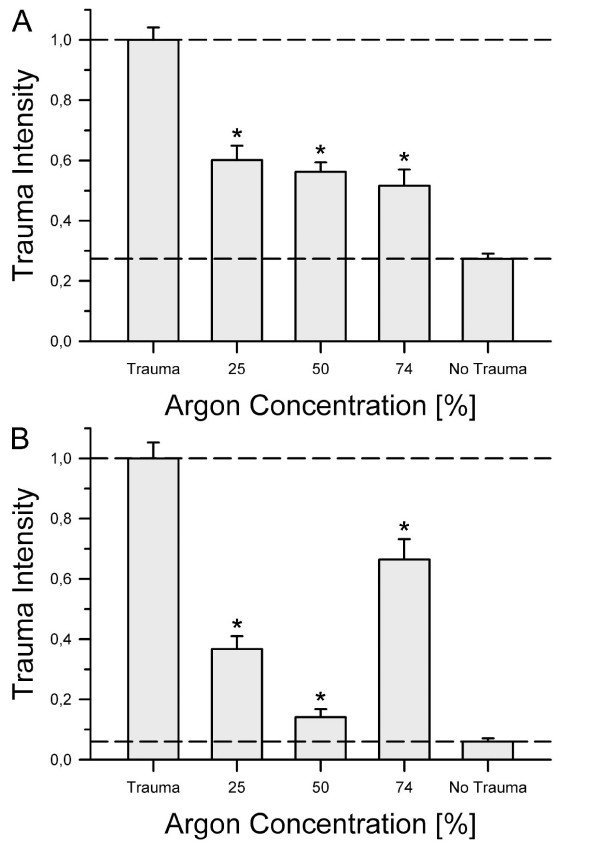
**Neuroprotective effects of argon**. Following trauma (OGD or TBI), slices were incubated for 72 hours in an atmosphere containing either x = 25, 50 or 74% argon in addition to 21% O_2_, 5% CO_2 _and 74-x% N_2_. After fluorescence imaging and image analysis all groups were normalized to their respective trauma control group at t = 72 hours. **Panel A **shows the results for OGD. For each group an average of 55 slices with a minimum of 42 slices was used (prepared from four to six mice). The trauma intensity in each argon group was significantly lower compared to the trauma control group (*P ≤ 0.001), while there was no significant difference amongst the different argon groups. **Panel B **shows the results for TBI. An average of 43 slices and a minimum of 35 slices was used for each group (prepared from four to eight mice). The detected trauma for each argon concentration was significantly lower compared to the control group (*P ≤ 0.001). Furthermore there was a significant difference between the three argon gas mixtures (*P *≤ 0.004 between 25% and 74% Argon and *P *≤ 0.001 between 50% and 74% argon).

TBI produced less absolute tissue damage than OGD due to the focal nature of this injury. Nevertheless the difference between traumatized and non-traumatized groups was still greater in TBI due to the very low damage found in non-traumatized TBI-slices. More than likely this can be attributed to a longer and more strenuous procedure during OGD. While there were only two medium changes necessary during TBI, OGD slices were subjected to three exchanges. Furthermore the pressure chamber had to be flushed twice for OGD, exposing to a certain extent the surface of the slices to dehydration. Therefore, the TBI control group reached only 6% of the total trauma while the OGD control group showed almost 27.3% of maximum trauma intensity. After establishing a valid measurement for tissue injury, we tested the effects of argon on traumatized slices. We treated groups of slices with 25%, 50% or 74% argon after TBI or OGD was induced. Figure 2 demonstrates that argon provided a significant protective effect in OGD as well as in TBI. After OGD, argon decreased tissue injury by at least 40%. Figure 2, panel A shows the relationship between argon concentration and damage reduction at 37°C. A concentration of 74% argon was most effective (0.52 ± 0.05), yet at concentrations of 25% (0.60 ± 0.05) or 50% (0.56 ± 0.03) a significant reduction of trauma was observed.

**Figure 3:**
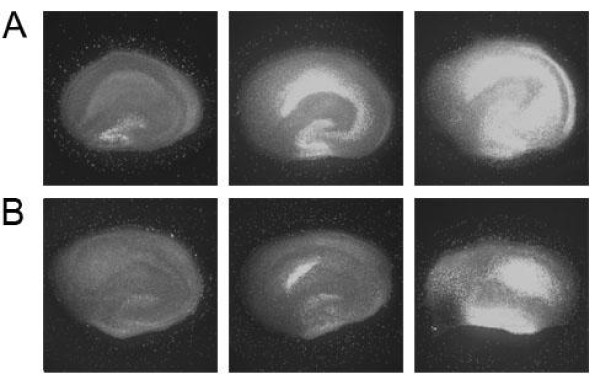
**Example images**. Panels **A **and **B **show example images for both OGD (panel A) and TBI (panel B). From left to right: No Trauma, 50% Argon, Trauma control.

Argon showed neuroprotective potential in TBI (Figure 2, panel B). The protection was most effective at a concentration of 50% (0.14 ± 0.03); however, it was still effective at 25% (0.37 ± 0.04) and 74% (0.66 ± 0.07) argon (see exemplary fluorescence images for traumatized and non-traumatized control slices and slices treated with 50% argon in Figure 3).

**Figure 4:**
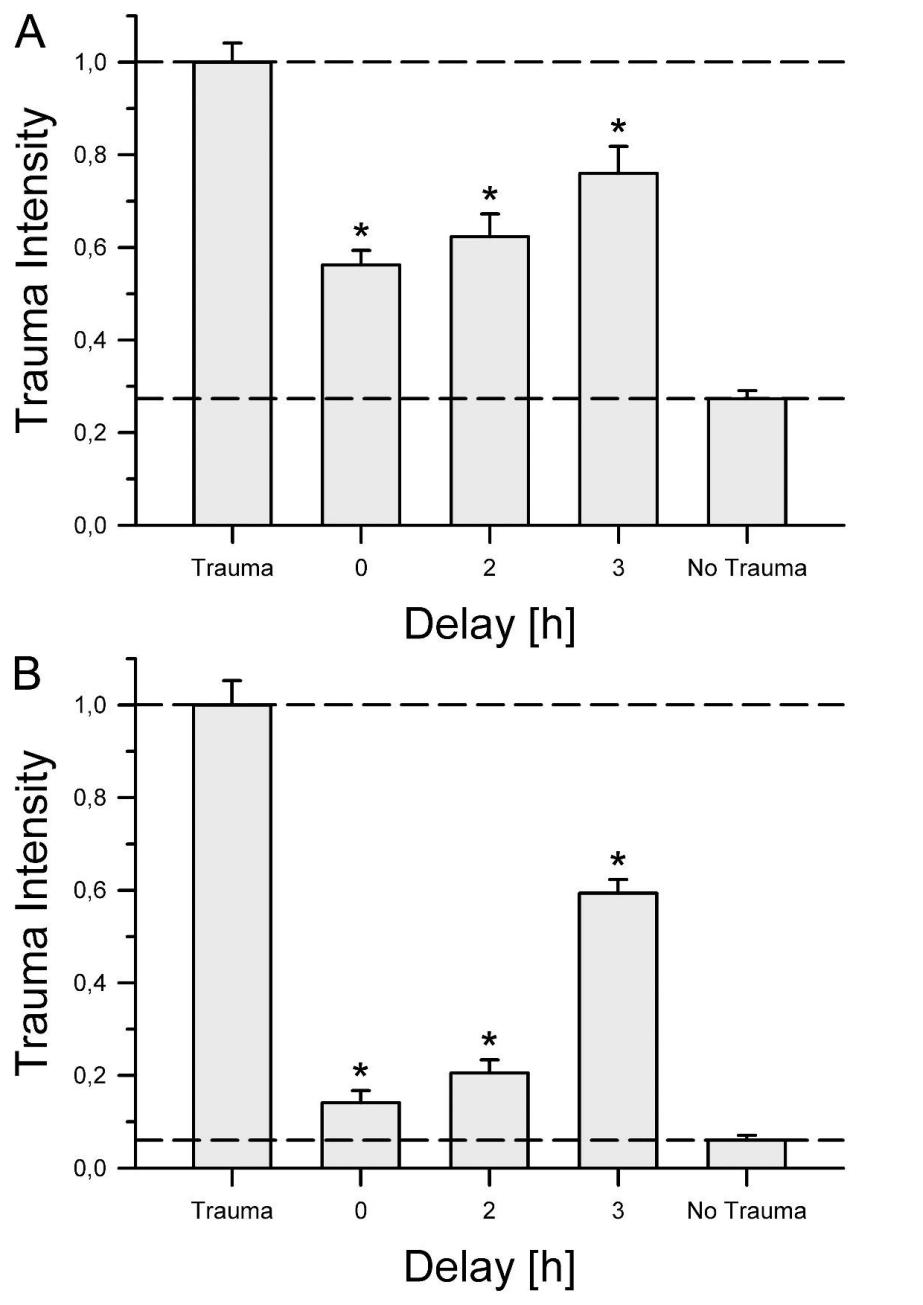
**Delayed argon application**. In this setting, groups were incubated for 72 hours in an atmosphere of 50% Argon, 21% O_2_, 5% CO_2 _and 24% N_2_, either directly after trauma was induced (t = 0) or with a two or three hours delay. All groups were normalized to their respective trauma control group at t = 72 hours. **Panel A **shows the results for OGD and **panel B **the results for TBI. In the OGD group an average of 43 slices with a minimum of 22 slices was used (prepared from three mice per group). We found a significant difference between the control group and each tested time point (*P ≤ 0.001). Moreover the trauma intensity between t = 0 hours and t = 3 hours differed significantly (*P *≤ 0.05). In the TBI group the detected trauma after zero, two and three hours delay time was significantly lower compared to the trauma control group (*P ≤ 0.001). Furthermore, trauma intensity after three-hour delay time was significantly increased as compared to zero and two-hour delay (*P *≤ 0.001). An average of 31 slices and a minimum of 15 slices was used for each group (prepared from two to three mice).

To adapt our laboratory setting more closely to a typical clinical situation, we decided to apply argon two and three hours after trauma. We incubated the slices in an atmosphere containing 50% argon because this concentration showed the best neuroprotective effect after TBI and there was no significant difference detectable between these three concentrations in OGD. As before, we could show that Argon strongly reduced cell damage in OGD and likewise in TBI (see Figure 4, panels A and B, respectively). Although there was an increase in tissue damage when argon application was delayed, argon was still significantly neuroprotective even two and three hours after injury.

## Discussion

We investigated the potential neuroprotective effects of the noble gas argon in two *in vitro *models of OGD and TBI. Our methods involved depriving cultured hippocampal slices of oxygen and glucose or producing a focal mechanical trauma on the CA1 region. Our data demonstrate argon's neuroprotective effect when it was applied directly as well as two and three hours after trauma.

Cultured organotypic hippocampal slices are a well established [27,28]* in vitro *model to gain easy access to nerve tissue. This model presents a reasonable compromise between dissociated cell cultures and models using intact living animals as most neuronal and glial cells survive [27] and their cytoarchitecture and connective organisation are well preserved [18,23,28-32]. Reducing the complex functions of the *in vivo *state to *in vitro *settings bears both benefits and disadvantages which have to be taken into account when interpreting our findings. However, this model mirrors to a certain extent the *in vivo *characteristics, when complicating systemic factors like blood pressure are excluded.

Utilization and outcome of OGD as a model of ischemia have proven to be very reproducible and are widely used [4,33-36]. While the complete pathogenic pathways of stroke are still incompletely understood, several mechanisms (including increased glutamate, calcium overload, mitochondrial dysfunction and oxidative stress) have been proposed to contribute to neuronal damage [28]. OGD, in contrast to other *in vitro *ischemic models such as glutamate excitotoxicity, might be more suitable to mimic this *in vivo *situation, as it allows for more than one pathomechanism elicited by energy depletion to occur. Although a wide range of possible neuroprotective compounds such as glutamate receptor antagonists [37], caspase inhibitors [38], anticonvulsants [39] and volatile anaesthetics [40] have been tested in an OGD setting, inert gases other than xenon have heretofore been scarcely investigated. Consequently, limited data are available on argon's organ-protective potential. Yarin [3] showed that argon protects rat's hair cells against ototoxic processes. In another rat model, Soldatov and co-workers [2] found that a gas mixture containing 25% argon improved the animals' survival under hypoxic conditions compared to a similar respiratory gas mixture without argon.

Jawad et al. [4] were the first investigators to show that 75% argon, administered during OGD and 24 hours thereafter, had neuroprotective effects. However, these results were limited to cultures of dissociated neurons. Therefore we used slice cultures in our study as a more complex and lifelike model. We could confirm argon's neuroprotective potential, even when administered after trauma. Furthermore, we could establish a concentration-dependent effect using three different argon concentrations. There was no significant difference in neuroprotective efficacy between the different argon concentrations in the OGD setting. However, there was a peak effect at 50% argon in the TBI-model. Interestingly, a similar observation about a peak effect of 50% xenon in the same *in vitro *model has been made by Coburn and colleagues. Yet, this was a theoretical assumption based on extrapolated data [8]. More importantly, with regard to typical clinical situations, we could demonstrate that argon significantly reduced neuronal damage even when applied two or three hours after OGD.

The possible effects of argon on TBI were completely unknown. Therefore we tested argon's impact in an *in vitro *model by inducing a focal mechanical trauma. This model has been widely used before by us and others when testing possible treatments [8,19,25] for traumatic brain injury. Nevertheless this is a simplified imitation of brain trauma, which lacks pathomechanisms involving systemic variables (for example, blood pressure) or local swelling, inflammation, ischemia and/or hypoxia. Yet, despite these obvious limitations, it approximates the *in vivo *situation thereby validating its clinical feasibility [41]. It is generally accepted that TBI damage is caused by two main factors. The initial lesion is mediated through direct mechanical damage at the impact site. Subsequently, several cellular and molecular processes expand the local injury. The so-called secondary injury is amongst others caused by excitotoxicity [42], up-regulation of cell-death genes [43], the formation of free radicals and the activation of pro-apoptotic mediator pathways [44-46].

Since medical intervention cannot rescue directly traumatized, dying cells, cells near the impact site surviving the initial assault are the main target for the neuroprotective potential of drugs [43]. Indeed, our experiments showed that argon was able to reduce cell death significantly, whether it was applied directly after the trauma or two and three hours afterwards. Of particular significance is argon's potential in protecting neuronal cells when argon administration was delayed. One of the many reasons why positive *in vitro *results do not transfer favourably to clinical trials [47] is that in many laboratory models treatment is only applied during the course of injury or directly thereafter. We decided to explore the outcome of delayed argon application solely with a gas mixture containing 50% argon for two reasons. First, in the TBI setting 50% argon was most effective. Secondly, and clinically more relevant, 50% of argon allows a higher inspiratory oxygen concentration for patients who require it.

Of consequence, especially in times of cost reduction, argon is the most abundant inert gas which is already widely used in other industries and therefore available at a relatively low price (nine cents/l) compared to xenon (20 €/l). Furthermore, argon has no anaesthetic properties at normobaric conditions [48]. It may therefore be used when sedation would be inappropriate.

While to date little is known about argon's mechanism of action, it has been proposed that argon triggers gamma-aminobutyric acid (GABA) neurotransmission by acting at the benzodiazepine binding site and possibly at multiple other discrete sites of the GABA_A _receptor [49]. The activation of GABA receptors has been shown to be neuroprotective in *in vitro *and *in vivo *ischemia models and several potential mechanisms have been proposed [40,50]. First, glutamatergic and GABAergic activity counterbalance the function of each other. On an electrochemical level, stimulation of the ionotropic GABA_A _receptor and the following chloride-based membrane hyperpolarization inhibit N-methyl-D-aspartate (NMDA) receptors [51]. In an *in vivo *rat model, Zhang et al. could show that GABA receptor activation diminished the phosphorylation of the NR2A subunit of the NMDA receptor, thus directly attenuating the receptors functionality [52]. NMDA receptor activation is generally seen as a key element in the development of neuronal death following ischemic events by, among others, increasing calcium influx [53,54]. Second, GABA receptor activation directly influences downstream pathogenic pathways. Galeffi et al. showed that diazepam counteracted ATP depletion and disabled cytochrome c release in rat hippocampal slices after OGD, two main events in the ischemic cascade [55]. Xu and co-workers demonstrated that GABA agonists inhibited pro-apoptotic pathways through activation of the phosphoinositide 3-kinase/protein kinase B cascade [56]. These findings can be a starting point for further studies to describe argons definite mode of action.

Nevertheless it is surprising that noble gases are able to generate an effect as it requires forming a chemical bond with another molecule. The gas molecules' outer valence shell is completely filled with electrons, making the gas inert to basic chemical reactions. However, these freely vacillating electrons can be polarized. Trudell et al. suggest that a charged element of the binding site itself can induce a bipole in the gas molecules, thus generating enough binding energy to form a bond with the binding site. Another component of the binding energy might be the London dispersion force which is generated by changes in electron density. When the distribution of electrons in one molecule fluctuates to produce an instantaneous dipole, this dipole can produce a dipole in a second molecule [57]. Thus, the previously uncharged gas molecules are temporarily polarized and therefore enabled to interact with the binding site.

## Conclusions

This study shows that argon bears surprisingly effective neuroprotective potential in both *in vitro *models of ischemia and traumatic brain injury. Protection was observed with three different concentrations of argon (25, 50 and 74%), either directly applied after the trauma or when administered at a concentration of 50% two and three hours after the injuries. Considering these promising results, despite the inherent simplifications of any *in vitro *model, further animal research, preferably using a whole animal model, seems appropriate.

## Key messages

• We found that Argon exerts neuroprotective effects in two different types of brain lesion (oxygen-glucose deprivation and traumatic brain injury) in organotypic hippocampal slice cultures when administered after trauma.

• Protection was observed with three different concentrations of argon (25, 50 and 74%).

• Even 3 hours after trauma, argon (50%) was still neuroprotective in both models of injury.

## Abbreviations

ANOVA: analysis of variance; ATP: adenosine triphosphate; EM: experimental medium; GABA: gamma-aminobutyric acid; NMDA: N-methyl-D-aspartate; OGD: oxygen-glucose deprivation; PI: propidium iodide; SEM: standard error of the mean; TBI: traumatic brain injury.

## Competing interests

MC and RR received lecture and consultant fees from Air Liquide Santé International, a company interested in developing clinical applications for medical gases, including argon and xenon. All other authors declare that they have no competing interests.

## Authors' contributions

PDL conducted the experimental laboratory work, performed the statistical analysis and drafted the manuscript. RR participated in the study design and coordination and helped to draft the manuscript. JR, JW, MF, AF, YR and OG helped to perform the study and draft the manuscript. MC conceived of the study, participated in the study design and coordination and helped to draft the manuscript. All authors read and approved the final manuscript.
